# Left parietal tACS at alpha frequency induces a shift of visuospatial attention

**DOI:** 10.1371/journal.pone.0217729

**Published:** 2019-11-27

**Authors:** Teresa Schuhmann, Selma K. Kemmerer, Felix Duecker, Tom A. de Graaf, Sanne ten Oever, Peter De Weerd, Alexander T. Sack

**Affiliations:** 1 Department of Cognitive Neuroscience, Faculty of Psychology and Neuroscience, Maastricht University, Maastricht, The Netherlands; 2 Brain Imaging Center, Maastricht, The Netherlands; 3 Department of Psychiatry and Neuropsychology, School for Mental Health and Neuroscience (MHeNs), Brain + Nerve Centre, Maastricht University Medical Centre+ (MUMC+), Maastricht, The Netherlands; University Medical Center Goettingen, GERMANY

## Abstract

**Background:**

Voluntary shifts of visuospatial attention are associated with a lateralization of parieto-occipital alpha power (7-13Hz), i.e. higher power in the hemisphere ipsilateral and lower power contralateral to the locus of attention. Recent noninvasive neuromodulation studies demonstrated that alpha power can be experimentally increased using transcranial alternating current stimulation (tACS).

**Objective/Hypothesis:**

We hypothesized that tACS at alpha frequency over the left parietal cortex induces shifts of attention to the left hemifield. However, spatial attention shifts not only occur voluntarily (endogenous/ top-down), but also stimulus-driven (exogenous/ bottom-up). To study the task-specificity of the potential effects of tACS on attentional processes, we administered three conceptually different spatial attention tasks.

**Methods:**

36 healthy volunteers were recruited from an academic environment. In two separate sessions, we applied either high-density tACS at 10Hz, or sham tACS, for 35–40 minutes to their left parietal cortex. We systematically compared performance on endogenous attention, exogenous attention, and stimulus detection tasks.

**Results:**

In the endogenous attention task, a greater leftward bias in reaction times was induced during left parietal 10Hz tACS as compared to sham. There were no stimulation effects in either the exogenous attention or the stimulus detection task.

**Conclusion:**

The study demonstrates that high-density tACS at 10Hz can be used to modulate visuospatial attention performance. The tACS effect is task-specific, indicating that not all forms of attention are equally susceptible to the stimulation.

## Introduction

Visual scenes typically include many stimuli. As our brains are not able to efficiently process all stimuli simultaneously, some need to be prioritized over others. Such prioritisation can be achieved through visuospatial attention, which enables preferential processing of stimuli at a location of interest [1]. To some extent, we naturally display preferential processing in one hemifield compared to the other [2]; we have a *spatial attention bias*. Furthermore, spatial attention can be shifted, either voluntarily (endogenous; top-down), or automatically, when captured by a salient stimulus (exogenous; bottom-up).

Various tasks can be used to assess attentional processes. *Visual detection tasks* require participants to detect low-contrast stimuli in the left, the right or both hemifields simultaneously and primarily assess low-level perceptual sensitivity. Detection tasks can reveal information about basic attentional biases, but they do not include an attentional manipulation. Therefore, these tasks leave higher-order attentional processes out of consideration. In contrast, *endogenous* and *exogenous attention tasks* directly capture higher-order attentional processes by manipulating the attentional focus with cues and measuring the subsequent target discrimination performance [3]. In endogenous attention tasks, centralized, symbolic cues predict the correct target location with high reliability and therefore motivate the participant to initiate endogenous (top-down) shifts of attention towards the cued location. Exogenous attention tasks, one the other hand, include salient lateralized cues that automatically attract rapid, exogenous (bottom-up) attention shifts, independently of the cue predictability. While exogenous attention shifts are stimulus-driven and therefore reflexive and fast [4,5], endogenous attention shifts require an interpretation of the symbolic cue and voluntary orienting of attention and are therefore relatively slow [4,5].

While it is easy for healthy participants to shift their attention, hemineglect patients show a pathological attention bias, marked by reduced responses to stimuli in the contralesional hemifield, generally caused by unilateral stroke in the temporoparietal lobe [6,7]. Hemineglect is more frequent and severe after right-hemispheric damage and is therefore commonly associated with a pathological rightward bias [7,8]. Patients are slower and less accurate in contralesional target detection [9,10], and display a spatial orienting bias in endogenous and exogenous tasks [11–13]. Especially in the context of multiple simultaneously presented stimuli, hemineglect patients often fail to detect stimuli in the contralesional hemifield and only pay attention to the ipsilateral hemifield, a form of a bias in attentional selection [8,14]. Electroencephalography (EEG) data shows that the amplitude [15–17] and amplitude variability [15] of alpha oscillations, which have been linked to attention, are reduced over their damaged hemispheres. In the stroke recovery period, alpha power increases again [18] and was shown to be associated with clinical improvement [17,19,20].

Numerous EEG studies with healthy participants suggest that alpha oscillations play an important role in information processing [21–23]. In attention research, it has been shown that a shift of endogenous attention is associated with a parieto-occipital alpha (8-12Hz) power lateralization [24–27]. This alpha power lateralization is characterised by an increase in oscillatory alpha power on the ipsilateral relative to the contralateral side of attention [24–27]. Thut and colleagues [7] report that alpha power lateralization can even be used to partially predict the momentary focus of spatial attention. More precisely, they found that the trial-by-trial variability in alpha power lateralization can be used to explain some variability in reaction times (RTs) to forthcoming lateralized target stimuli [27]. Interestingly, while endogenous attention shifts are associated with a lateralization of parieto-occipital alpha power, evidence for a similar link between exogenous attention and alpha power lateralization is lacking. The reason for the absence of evidence could lie in the temporal dynamics of exogenous attention shifts. As reflexive and rapid attentional process, it is methodologically difficult to reliably estimate alpha power with EEG during exogenous attention shifts. This limitation could be overcome using alternative techniques such as transcranial alternating current stimulation (tACS).

To empirically demonstrate the causal relevance of parietal alpha oscillations in attention, one should modulate alpha power experimentally. This can be achieved with non-invasive brain stimulation (NIBS) techniques such as tACS. tACS consists of low-intensity electrical current flowing rhythmically back and forth between two (or more) electrodes [28–30]. Recent studies combining tACS with EEG show that tACS at alpha frequency leads to an elevation of alpha power [29–32]. tACS has been used to study the functional role of oscillations in various cognitive processes in healthy volunteers [33–38]. Unilateral tACS, tACS applied only to one hemisphere, at alpha frequency has previously been shown to influence spatial attention performance [37,38]. Left temporocentral alpha tACS has been shown to induce a leftward bias in an auditory attention/working memory task [37]. In the visual domain, right parietal alpha tACS modulated visuospatial attention performance in a landmark task (experiment 1, [38], although this finding could not be replicated (experiment 2, [38]).

In the current study, we aimed at investigating whether left posterior parietal tACS at alpha frequency results in a shift of visuospatial attention to the left hemifield in healthy volunteers. We were particularly interested in targeting the left posterior parietal cortex because of the clinical potential of this intervention. If successful, the same procedure could be used to alleviate the pathological attentional rightward bias in hemineglect patients. We used a high-density ring electrode montage over the left posterior parietal cortex (PPC), an area that has shown to play an important role in the direction of attention towards a location of interest [39,40]. Considering the conceptual difference between low-level detection, endogenous and exogenous attention, we evaluated whether alpha tACS affected these processes differentially.

To address our research aims, we stimulated the left parietal cortex at alpha frequency (10Hz) and sham in separate sessions and measured visuospatial attention performance in a visual detection, endogenous attention, and exogenous spatial attention task. We hypothesized that left posterior parietal 10Hz tACS induces an attentional leftward bias relative to sham in the endogenous attention and detection task. As it is still unknown whether parietal alpha oscillations are also associated with exogenous attention shifts we had no a priori hypothesis regarding the effect of tACS on the exogenous attention task.

## Methods

### Participants

The study was approved by the Ethics Review Committee Psychology and Neuroscience, Maastricht, Netherlands. All participants gave written informed consent prior to participation. We tested 36 healthy, right-handed students with normal or corrected to normal vision (18 women, mean age = 21.56 years, age range = 18–29 years). At the beginning of each session, participants gave their written informed consent and were screened for tACS safety. We followed the recommended safety procedures of Antal and colleagues [41], screening for e.g. skin diseases, neurological disorders, implants, pregnancy and medication.

### Procedure

Each participant took part in two sessions, an active 10Hz tACS session and a sham 10Hz tACS session. Experimental sessions were minimally two days apart and the order of sessions was randomized. Each session started with practicing the detection, endogenous attention and exogenous attention task. tACS was subsequently applied at either 10Hz or sham during which participants performed the visuospatial attention tasks. In every session, participants performed all three visuospatial attention tasks in a counterbalanced order. tACS never exceeded 40 minutes and was switched off after completion of all tasks. An eye tracker was used for the endogenous and exogenous attention task during tACS to record eye movements. Participants were blind to the stimulation protocol and the experimental hypotheses. A post-questionnaire was used at the end of each session to assure that blinding was maintained.

### tACS and electric field simulation

**Fig 1:**
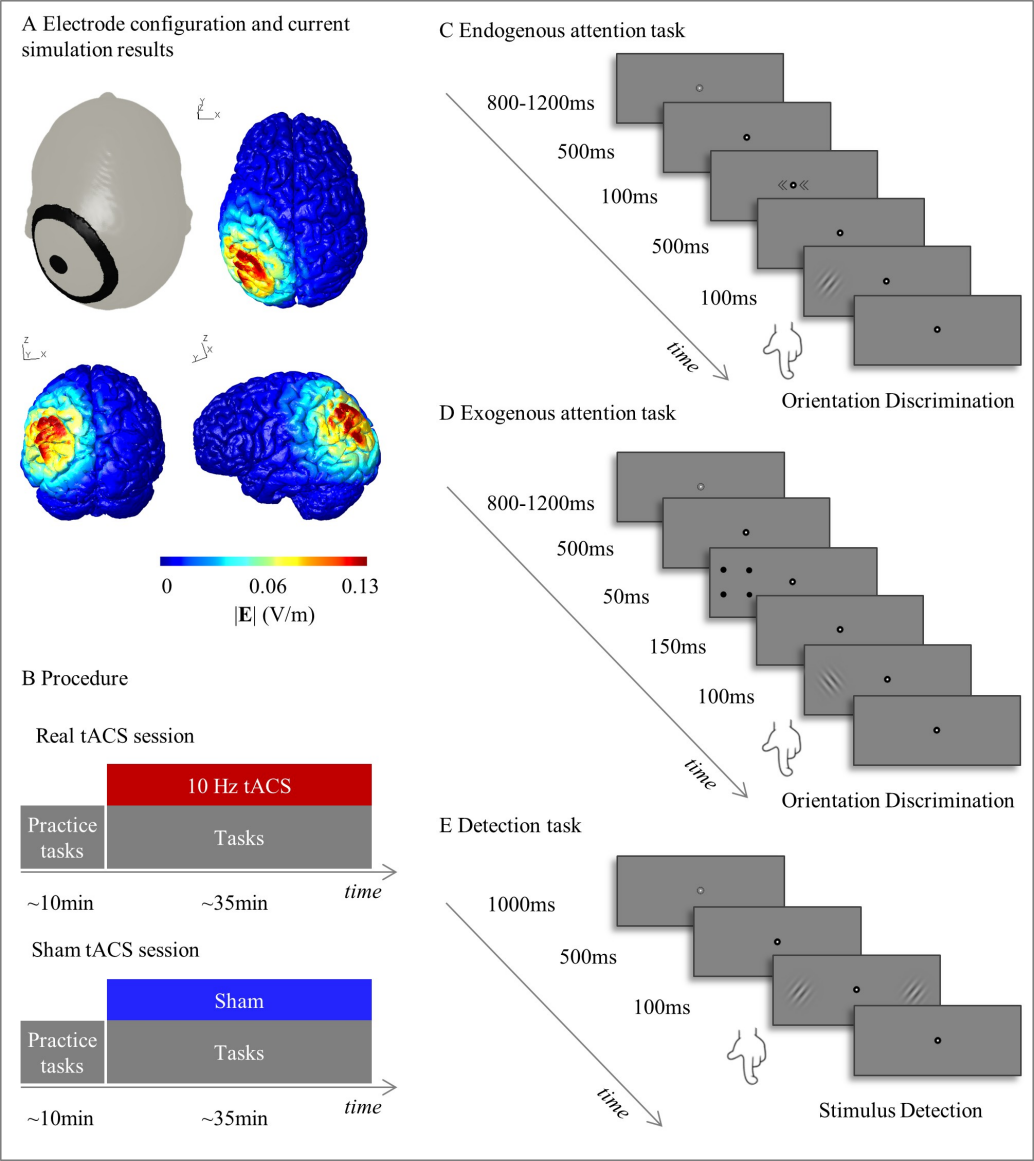
Experimental setup, procedure and example trials from the three attention tasks. (A) *tACS electrode configuration and current simulation results*. tACS ring electrodes were centered on the left posterior parietal cortex (P3). The results of a current simulation using the software SimNIBS depict the norm of the electric field (V/m) on an example brain from a transverse, coronal and left sagittal view. (B) *Procedure*. Each participant went through a real and a sham tACS session, which took place on separate days. Every session started with a short practice version of the attention tasks. Afterwards, participants performed the complete version of the tasks while being stimulated at either 10Hz or sham. C) *Example trial from the endogenous attention task*. A trial started with the presentation of a fixation point followed by an endogenous cue (<<**•**<<, >>**•**>> or <<**•**>>), which informs the participant to direct voluntary, endogenous attention to the left, right or both sides. Thereafter, a sinusoidal grating tilted 45° to either side was shown in the left or right hemifield. The participants had to indicate whether the grating was turned to the left or right by pressing the corresponding button (valid trial in this example). (D) *Example trial from the exogenous attention task*. Similarly to the endogenous attention task, a trial started with the presentation of a fixation point followed by an exogenous cue. The exogenous cue consisted of four black dots forming a square and surrounding either the left or right potential Target Location (directional cue) or a luminance change of the background color of the screen (neutral cue). As opposed to the endogenous version, this exogenous cue triggers a sudden shift of exogenous attention. Afterwards, the sinusoidal grating was presented in the left or right hemifield and participants had to discriminate its orientation (invalid trial in this example). (E) *Example trial from the detection task*. A sinusoidal grating was presented either in the left, right or both hemifields. Participants had to indicate the location of the sinusoidal grating (bilateral target in this example).

We stimulated the left PPC with a high-density ring electrode montage. A small circular (diameter: 2.1cm, thickness: 2mm) and a large (Outer diameter: 11cm; Inner diameter: 9cm, thickness: 2mm) rubber ring tACS electrode (NeuroConn, Ilmenau, Germany) were placed onto the left parietal cortex. The small electrode was positioned over P3, localized based on the international 10–20 EEG system and the large electrode was centred on it (Fig 1A). Conductive gel (Ten20 paste, Weaver and Company, Aurora, CO, USA) was applied between skin and electrodes to reduce the impedance to below 10kΩ. Stimulation frequency and intensity were set to 10Hz and 1mA peak to peak, phase offset was set to 0 and 100 cycles were used for ramping up. The sham stimulation also consisted of tACS at 10Hz but was ramped up and then immediately ramped down with each 100 cycles.

This ring electrode montage enables a higher spatial focality as compared to standard rectangular electrodes [42]. We ran an electric current simulation to visualize which regions were stimulated using the ring electrode configuration centered on P3 (Fig 1A). The simulation was done using a custom-written MATLAB script [43] interfacing with the software SimNIBS [44,45]. As an example participant we used a freely available individual head model of a healthy brain [46]. The electrodes were modelled with a random connector location and the conductivity of the ten20 paste was set to 8 S/m (estimation is based on the concentration of Cl^-^ in the gel [47]).

### Task description

#### Endogenous attention task

In the endogenous attention task, participants had to perform an orientation discrimination task with central symbolic cues prompting top-down covert shifts of attention to the cued location. At all times, a white fixation point surrounded by a grey or black area, delimited by a black circle was continuously presented at the center of the screen. The endogenous attention task started with a jittered interval of 800-1200ms. Afterwards, the grey area surrounding the fixation turned black for 500ms, alerting the participant that stimuli will soon appear. Subsequently, an endogenous symbolic cue appeared for 100ms. The cue consisted of two arrowheads pointing to the left (<<•<<), right (>>•>>) or both sides (<<•>>) flanking the fixation point and predicting the correct Target Location with 80% validity. The arrowheads pointing to the left or right hemifield are called directional cues as they inform the participant to allocate (endogenous) attention towards either hemifield. The arrowheads pointing towards both sides serve as neutral cue as they encourage the participant to equally distribute the attention over both hemifields. After presentation of the cue, only the fixation point was shown for 500 ms. Subsequently a sinusoidal grating with a Gaussian envelope appeared at 7° eccentricity (angular distance from the center of the visual field) from the fixation point either in the left or right hemifield (Fig 1C) for 100ms. The grating was rotated by 45° in either counter-clockwise or clockwise direction and the task of the participant was to discriminate its orientation by pressing the button with number 1 or 2 respectively as quickly as possible. For both the endogenous as well as the exogenous attention task, the three Type of Cues (left, right, neutral) and the two Target Locations (left, right) result in six different experimental conditions. The cues gave rise to valid and invalid trials, depending on whether the Target Location matched the cued location (valid) or not (invalid). In the neutral condition, spatially uninformative cues are presented followed by a target stimulus in either hemifield. The endogenous attention task consisted of 336 trials in total, divided into 192 valid, 48 invalid, and 96 neutral cue trials and task duration was approximately 20 minutes.

#### Exogenous attention task

In the exogenous attention task, participants again had to perform an orientation discrimination task, this time with peripheral cues prompting bottom-up covert shifts of attention to the cued location. A white fixation point surrounded by a grey or black area and delimited by a black circle was presented for a jittered interval of 800-1200ms. Subsequently, the grey area turned black for 500ms (Fig 1D). Then an exogenous, salient cue was presented peripherally for 100ms, triggering a sudden shift of (exogenous) attention. The exogenous cues consisted of either four small dots surrounding a potential Target Location (directional cues with 50% validity) or a luminance change of the background color of the screen (neutral cues). The cues were presented for 50ms followed by a cue-target interval of 150ms. Afterwards, a sinusoidal grating with Gaussian envelope appeared at 14° eccentricity from the fixation point in either hemifield. As in the endogenous versions, the participant had to discriminate its orientation by pressing the button with number 1 for counter-clockwise and button with number 2 for clockwise rotated targets as quickly as possible. The exogenous attention task consisted of 216 trials in total with 72 trials per *Type of Cue* (valid, neutral, invalid), lasting approximately 10 minutes in total.

#### Detection task

In the detection task, participants had to detect low-contrast stimuli in the left, the right or both sides of the fixation point. The task was preceded by a short calibration procedure during which bilateral stimuli were presented peripherally, on the same location as presented in the actual task and participants manually down-regulated the contrast of the stimulus to slightly above their detection threshold. This value was used as an initial contrast value for the detection task. The detection task then started with the presentation of a grey fixation point for 1000ms, which then changed in grey-scale for 500ms to indicate the start of the trial. Thereafter, sinusoidal gratings (spatial frequency = 1.5 cycles per degree, envelope standard deviation = 0.75°) with random orientation were presented in the left, right, or both hemifields for 100ms at 14° eccentricity (Fig 1B). Participants were instructed to indicate the location of the stimulus by pressing the button with number 1, 2 and 3 for left, bilateral and right Target Locations respectively and to withhold their response in case the participants did not perceive a stimulus at all. The contrast of the stimuli in the left, right or both hemifields was adapted for each of the locations separately on a trial-by-trial basis according to the QUEST staircase algorithm aiming at 50% accuracy (prior standard deviation = 1, beta = 3.5, gamma = 0.01, delta = 0.01) [48]. QUEST, as implemented in the Psychophysics Toolbox [49] for MATLAB, is a Bayesian adaptive psychometric procedure, which uses information about the individual psychometric curve to guide further testing [48]. The initial mean was based on the short manual calibration procedure and the successive contrast values were adjusted according to QuestQuantile, which calculates the successive contrast after each trial based on the maximum likelihood estimate of threshold. The final detection threshold was estimated using QuestMean. One iteration of the detection task comprised three staircases of each 40 trials resulting in three contrast thresholds per session (one for left, one for right and one for the bilateral targets). In total, the detection task took approximately 10 minutes. Reaction times were recorded with a standard USB-computer keyboard and participants always used the right hand for the response.

All tasks were presented with 60Hz on a gamma-corrected liyama ProLite monitor at 57-cm viewing distance. Video mode was 1920x1080 and the background luminance was 100cd/m^2^. The software application presentation (NeuroBehavioural Systems, Albany, CA) was used for running the tasks.

### Eye tracker

Eye tracking (Eyelink1000, SR Research, Mississauga, Ontario, Canada) was used during the endogenous and exogenous cueing task. Initially a 9-point calibration and validation procedure was executed, followed by monocular eye tracking at 1000Hz to track gaze position sample by sample point. Participants were told to keep their chin in the chin rest at all times to avoid movements. Post-hoc trials containing eye blinks and eye movements exceeding 2° of visual angle in the time window from 100ms before cue onset until stimulus onset were deleted (5.92% of all trials for the endogenous and 2.65% for the exogenous version). No eye tracking was used for the detection task, as it does not include a (re)orienting component.

### Statistical analysis

#### Endogenous and exogenous attention task

For both the endogenous and exogenous attention tasks trials with extreme RTs based on the median +/- 1.5*interquartile range (IQR) criterion were removed. To assure a sufficient number of trials per cell the average amount of trials over both *Target Locations* (left, right) per *Stimulation Condition* (10 Hz tACS, sham) and *Type of Cue* (valid, neutral, invalid) was calculated. Only participants with more than 15 trials per Stimulation Condition and Type of Cue were included in the analysis.

We performed repeated measures analyses of variance (repeated measures ANOVA) to compare the condition averages of median RTs. A repeated measures ANOVA on sham tACS data, with factor Type of Cue, was performed to validate the attention tasks. For the endogenous and exogenous attention task, median RTs based on only correct trials were computed per Target Location, Type of Cue and Stimulation Condition. The reaction bias score was calculated by subtracting the RT to right from the RT to left targets. Resulting attention bias scores (reaction time bias) were compared in a repeated measures ANOVA with factors Type of Cue and Stimulation Condition. In a post-hoc analysis, we collapsed the median RTs across the three levels of cue and ran a repeated measures ANOVA with Target Location and Stimulation Condition as factors. The difference in RTs to targets in the left and right hemifield in the active 10Hz Stimulation Condition relative to the sham condition was tested with a t-test.

#### Detection task

In the detection task, we analyzed whether tACS at 10Hz induced a low-level detection performance bias by running a repeated measures ANOVA with *Stimulation Condition* (10 Hz tACS, sham) and *Target Location* (left, right, bilateral) as factors and contrast thresholds as dependent variable. The incorrect responses in bilateral trials were analyzed separately to investigate whether tACS affected attentional selection in the context of multiple simultaneously presented stimuli. We hypothesized that tACS at 10Hz induces a leftward bias in attentional selection, which means that participants would tend to perceive only the left instead of both lateralized targets. We therefore performed a repeated measures ANOVA with Stimulation Condition and *Indicated Target Location in Bilateral Trials* (left, right) as factors and number of trials as dependent variable. For comparability between tasks, we also post-hoc explored the differences in reaction time bias in the detection task. It should, however, be noted that participants were not explicitly instructed to respond as fast as possible and we therefore want to emphasize that the results should be interpreted with caution. For the analysis of the reaction time bias, we ran a paired samples t-test with Stimulation Condition (10Hz, sham) as factors and reaction time bias (RTleft targets—RTright targets) as dependent variable. All statistical tests were corrected for family-wise error rate using Bonferroni correction. Matlab R2016a was used for deriving the dependent variables and SPSS version 24 for running the statistics.

## Results

In the endogenous attention task, average accuracy was 93% (range: 69%-100%). Two participants were not included in the analysis because of an insufficient number of correct trials without eye artefacts. In the exogenous attention task, average accuracy was 93% (range: 67%-100%) and one participant had to be excluded from the analysis due to an insufficient number of correct trials without eye artefacts. All participants were included in the analysis of the visual detection task.

### Cueing effects in the endogenous and exogenous attention task

**Fig 2:**
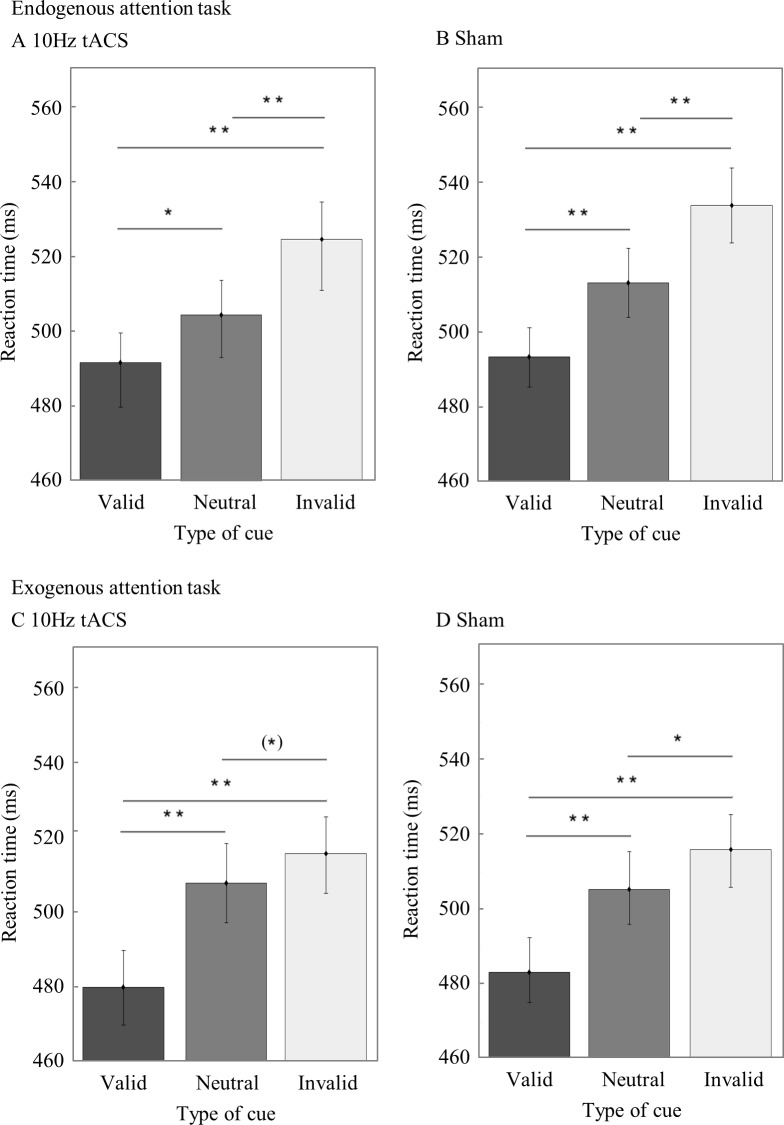
Cueing effect in the endogenous and exogenous attention task. RTs averaged over both *Target Locations* per Type of Cue for the endogenous attention task for the 10Hz tACS (A) and sham condition (B) as well as for the exogenous attention task for the 10Hz (C) and sham (D) condition. One asterisk visualizes a significant difference with a p-value < .05, a double asterisk stands for a p-value < .001, and an asterisk in brackets indicates a marginally significant effect.

To check whether the manipulation of attention with the endogenous cues was successful, we ran a repeated measures ANOVA with median RT averaged over both *Target Locations* (left target, right target) as dependent variable and *Type of Cue* (valid, neutral, invalid) as factor on the data of the sham session. Establishing the cueing effect we found a main effect of Type of Cue (F_2,66_ = 35.33, p<0.001, ηp^2^ = .52), with significantly slower RTs in invalid cue trials (M = 534.15, SEM = 10.02) as compared to neutral (M = 513.32, SEM = 9.25) (t_33_ = 4.23, p < .001, r^2^ = 0.35) and valid cue trials (M = 493.35, SEM = 7.95) (t_33_ = 7.04, p < .001, r^2^ = 0.60) and significantly faster RTs in valid as compared to neutral cue trials (t_33_ = -5.58, p < .001, r^2^ = 0.49) (Fig 2B). When repeating this analysis for the 10Hz tACS condition, we again found a significant cueing effect (F_2,66_ = 22.61, p < .001, ηp^2^ = .41), with slower RTs in invalid (M = 524.71, SEM = 13.62) as compared to neutral (M = 504.79, SEM = 11.34) (t_33_ = 3.89, p < .001, r^2^ = .31) and valid cue trials (M = 491.79, SEM = 11.99) (t_33_ = 5.77, p < .001, r^2^ = .50) and faster RTs in valid as compared to neutral cue trials (t_33_ = -3.46, p = .001, r^2^ = .27) (Fig 2A).

We performed the same analyses for the exogenous attention task. A main effect of Type of Cue (F_2,68_ = 29.58, p < .001, ηp^2^ = .47) with faster RTs in valid (M = 482.75, SEM = 9.30) as compared to neutral cue (M = 504.98, SEM = 10.16) trials (t_34_ = -5.97, p < .001, r^2^ = .51), faster RTs in valid as compared to invalid (M = 515.70, SEM = 9.42) cue trials (t_34_ = -6.88, p < .001, r^2^ = .58) and a significant difference between neutral and invalid cue trials (t_34_ = -2.37, p = .024, r^2^ = 0.14) (Fig 2D) was found. In the 10Hz tACS data, a significant cueing effect (F_2,68_ = 37.73, p < .001, ηp^2^ = .53) was found. Participants were significantly slower for invalid (M = 513.06, SEM = 10.01) as compared to valid cue trials (M = 479.18, SEM = 9.57) (t_34_ = 7.83, p < .001, r^2^ = .64) and faster in valid as compared to neutral cue trials (t_34_ = -6.20 p < .001, r^2^ = .53). The difference between invalid and neutral cue trials (M = 505.63, SEM = 10.10) was only marginally significant (t_34_ = 2.02, p = .051, r^2^ = .11) (Fig 2C).

### Reaction time bias in the endogenous attention task

**Fig 3:**
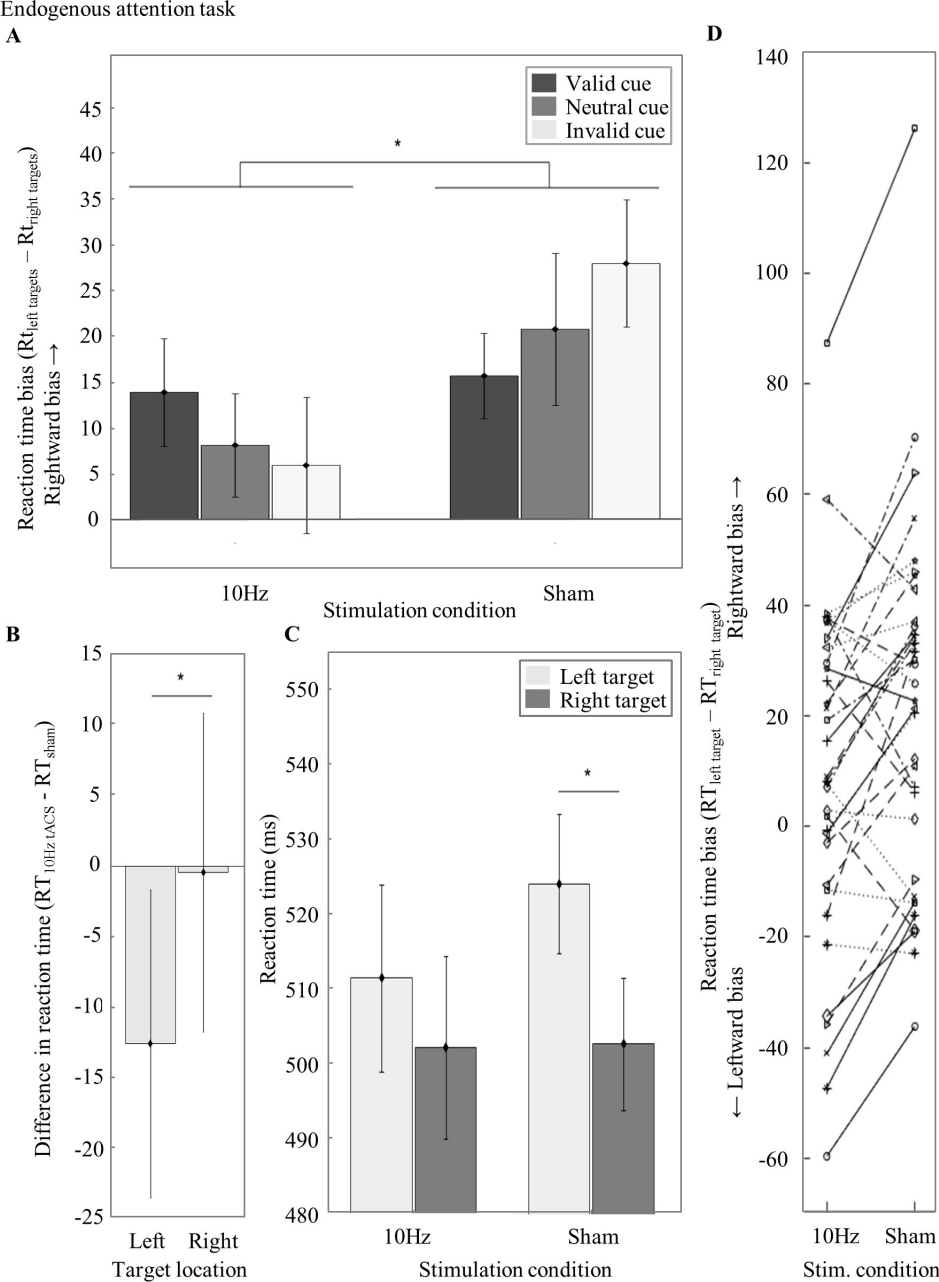
Results from the endogenous attention task. (A) *Reaction time bias in the endogenous attention task during 10Hz and sham for valid*, *neutral and invalid cue trials*. A positive value of reaction time bias (RTleft target−RT_right target_) indicates a rightward bias whereas negative values indicate a leftward bias of visuospatial attention. There is a significantly greater leftward bias during 10Hz tACS as compared to sham. (B) *Difference in reaction time per Target Location*. For 10Hz tACS relative to sham there is a difference in RTs for stimuli in the left (RTleft target, 10Hz tACS—RT_left target, sham_) as compared to the right hemifield (RTright target, 10Hz tACS—RT_right target, sham_). This means that we induced a leftward bias during 10Hz tACS relative to sham. (C) *RTs averaged over Type of Cue per Stimulation Condition and Target Location*. For the sham Stimulation Condition, participants reacted faster in response to stimuli in the right as compared to the left hemifield. This effect is attenuated for the 10Hz Stimulation Condition. (D) *Reaction time bias per participant for the 10Hz tACS and Sham Stimulation Condition*. Each line depicts the results of one participant. Error bars visualize the standard error of the mean (SEM) across participants. One asterisk visualizes a significant difference with a p-value < 0.05 and a double asterisk indicates a significant difference with a p-value < 0.001.

We analyzed whether left parietal alpha tACS shifts attention to the left hemifield, by evaluating the effects of *Stimulation Condition* (tACS, sham) and *Type of Cue* (valid, neutral, invalid) on reaction time bias (RT_left target−_RT_right target_) in a repeated measures ANOVA. There was a main effect of Stimulation Condition (F_1,33_ = 12.33, p = .001, ηp^2^ = .27) with a greater leftward bias (M = 9.29, SEM = 6.30) in the 10Hz as compared to the sham condition (M = 21.44, SEM = 6.61) (Fig 3A). The main effect of Type of Cue (F_2,66_ = .24, p = .790, ηp^2^ = .01) and the interaction effect were not significant (F_2,66_ = 2.21, p = .118, ηp^2^ = .06). Finding a main effect of Stimulation Condition confirmed our hypothesis that left parietal tACS at alpha frequency induces a leftward bias in visuospatial attention relative to sham. Another way to present the same results is to subtract the sham (baseline) session data from the 10Hz tACS session data (RT10Hz tACS—RT_sham_) and subsequently compare the difference in RTs between the two *Target Locations* (left, right) (Fig 3B; statistically, this is identical to the main effect of Stimulation Condition). Here it can be seen that participants are faster for stimuli in the left (M = -12.58, SEM = 10.94) as compared to the right hemifield (M = -.43, SEM = 11.32).

**Fig 4:**
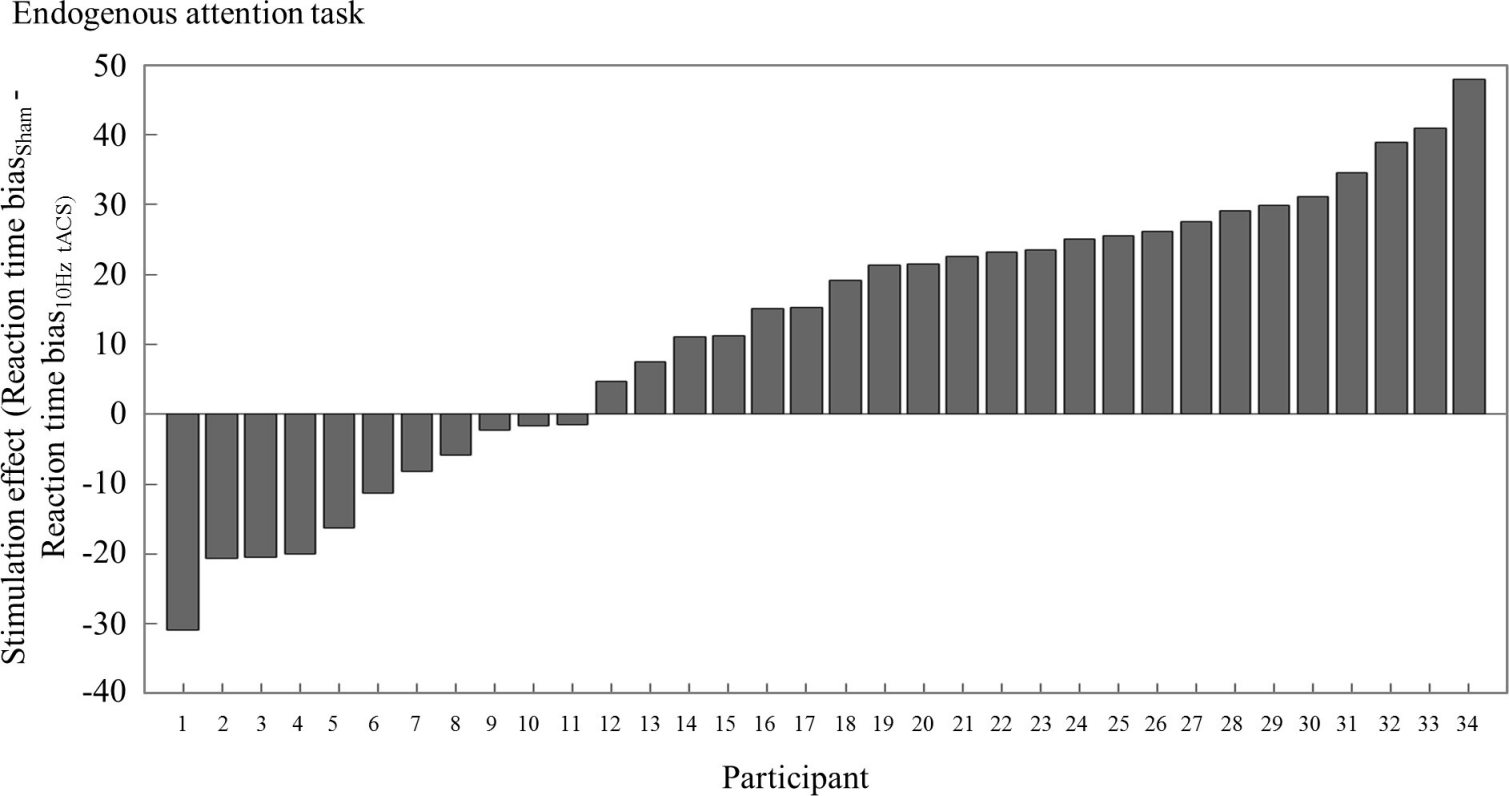
Behavioral stimulation effect per participant. (A) Each bar depicts the stimulation effect (reaction time bias_sham_—reaction time bias_10Hz tACS_) of one participant. A positive value means that the tACS intervention induced the expected behavioral effect in the endogenous attention task, i.e. a greater attentional leftward bias in the 10Hz tACS Stimulation Condition as compared to sham.

To see which Target Location drives the attentional bias effect, we analyzed the RTs averaged over all cues per Target Location and Stimulation Condition in a repeated measures ANOVA. There was no main effect of Stimulation Condition (F_1,33_ = .35, p = .558, ηp^2^ = .01), a main effect of Target Location (F_1,33_ = 8.64, p = .006, ηp^2^ = .21) and an interaction effect (F_1,33_ = 12.34, p = .001, ηp^2^ = .27). In follow-up t-tests we found slower RTs for the left (M = 524.41, SEM = 9.37) as compared to the right targets (M = 502.88, SEM = 8.88) for the sham Stimulation Condition (t_33_ = 3.81, p = .002, r^2^ = 0.31). In contrast, the left Target Location (M = 511.82, SEM = 12.50) did not differ from the right Target Location (M = 502.56, SEM = 12.17) for the 10Hz Stimulation Condition (t_33_ = 1.72, p = .376, r^2^ = 0.08). The left Target Location of the 10Hz Stimulation Condition did not significantly differ from the left Target Location in the sham Stimulation Condition (t_33_ = -1.15, p = 1.0, r^2^ = .04). Likewise, the right Target Location of the 10Hz Stimulation Condition did not differ from the right Target Location of the sham Stimulation Condition (t_33_ = -.04, p = 1.0, r^2^ < .01) (Fig 3C). An analysis of the reaction time bias per participant revealed that 23 out of 34 participants show the expected behavioral effect, with a greater leftward bias during 10Hz tACS as compared to sham (Figs 3D and 4).

We thus found a greater leftward bias in the 10Hz as compared to sham Stimulation Condition in the endogenous attention task, confirming our hypothesis. Follow-up analyses revealed that the RTs for neither the left nor right Target Location differed between the Stimulation Conditions. In the sham condition, there was a significant difference between the two Target Locations with faster RTs to stimuli in the right as compared to the left targets. In the 10Hz condition, the two Target Locations did not differ from each other. However, when subtracting the data of the sham from the 10Hz condition, we found a leftward bias with faster RTs for left as compared to right targets.

### Reaction time bias in the exogenous attention task

**Fig 5:**
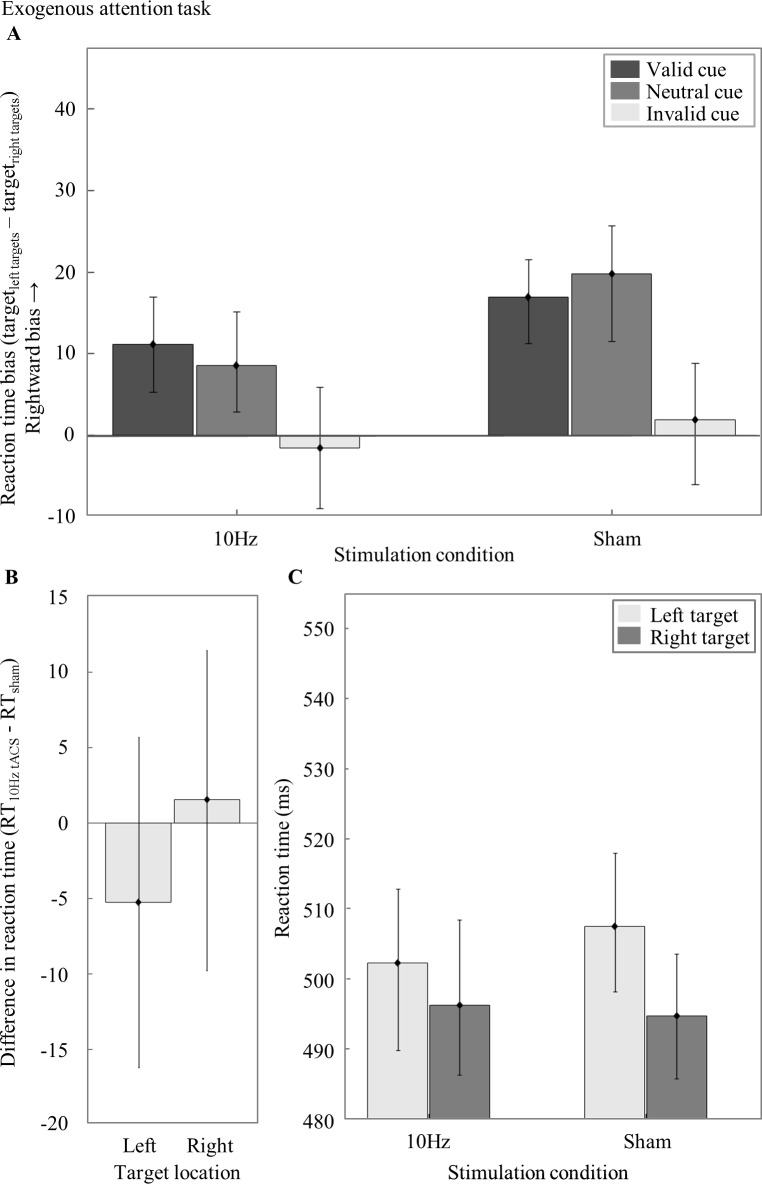
Results from the exogenous attention task. A) *Reaction time bias in the exogenous attention task for the 10Hz tACS and sham Stimulation Condition for valid*, *neutral and invalid cue trials*. (B) *Difference in reaction time (RT*_*10Hz tACS*_*—RT*_*sham*_*) per Target Location*. (C) *RTs averaged over all Type of Cues per Stimulation Condition and Target Location*. Error bars depict the standard error of the mean (SEM) across participants. A single asterisk visualizes a significant difference (p-value < 0.05) and a double asterisk indicates a highly significant difference (p-value < 0.001).

The effect of 10Hz tACS on visuospatial attention relative to sham in the exogenous attention task was investigated with a repeated measures ANOVA with reaction time bias (RT_left targets_—RT_right targets_) as dependent variable and *Stimulation Condition* (10Hz, sham) and *Type of Cue* (valid, neutral, invalid) as factors. There was a main effect of Type of Cue (F_2,68_ = 6.61, p = .002, ηp^2^ = .16) but neither a main effect of Stimulation Condition (F_1,34_ = 2.25, p = .143, ηp^2^ = .06) nor an interaction effect (F_2,68_ = .50, p = .610, ηp^2^ = .01) (Fig 5A). This means that tACS at 10Hz did not induce a significant leftward bias relative to sham.

A more intuitive way of presenting the results of the same analysis is to subtract the sham (baseline) session data from the 10Hz tACS session data and subsequently compare the RTs between the left (M = -5.27, SEM = 10.97) and the right (M = 1.56, SEM = 9.91) *Target Location* (Fig 5B; statistically identical to the main effect of Stimulation Condition).

Similar to the analysis of the endogenous attention task, we analyzed the RTs averaged over all cues per Target Location and Stimulation Condition in a repeated measures ANOVA. There were no significant effects (Stimulation Condition: (F_1,34_ = .03, p = .857, ηp^2^ < .01), Target Location: (F_1,34_ = 3.34, p = .077, ηp^2^ = .09), Stimulation Condition * Target Location: (F_1,34_ = 2.25, p = .143, ηp^2^ = .06) (Fig 5C). Hence, 10Hz stimulation did not significantly affect visuospatial attention performance in the exogenous attention task

### Contrast threshold bias and error bias in the detection task

**Fig 6:**
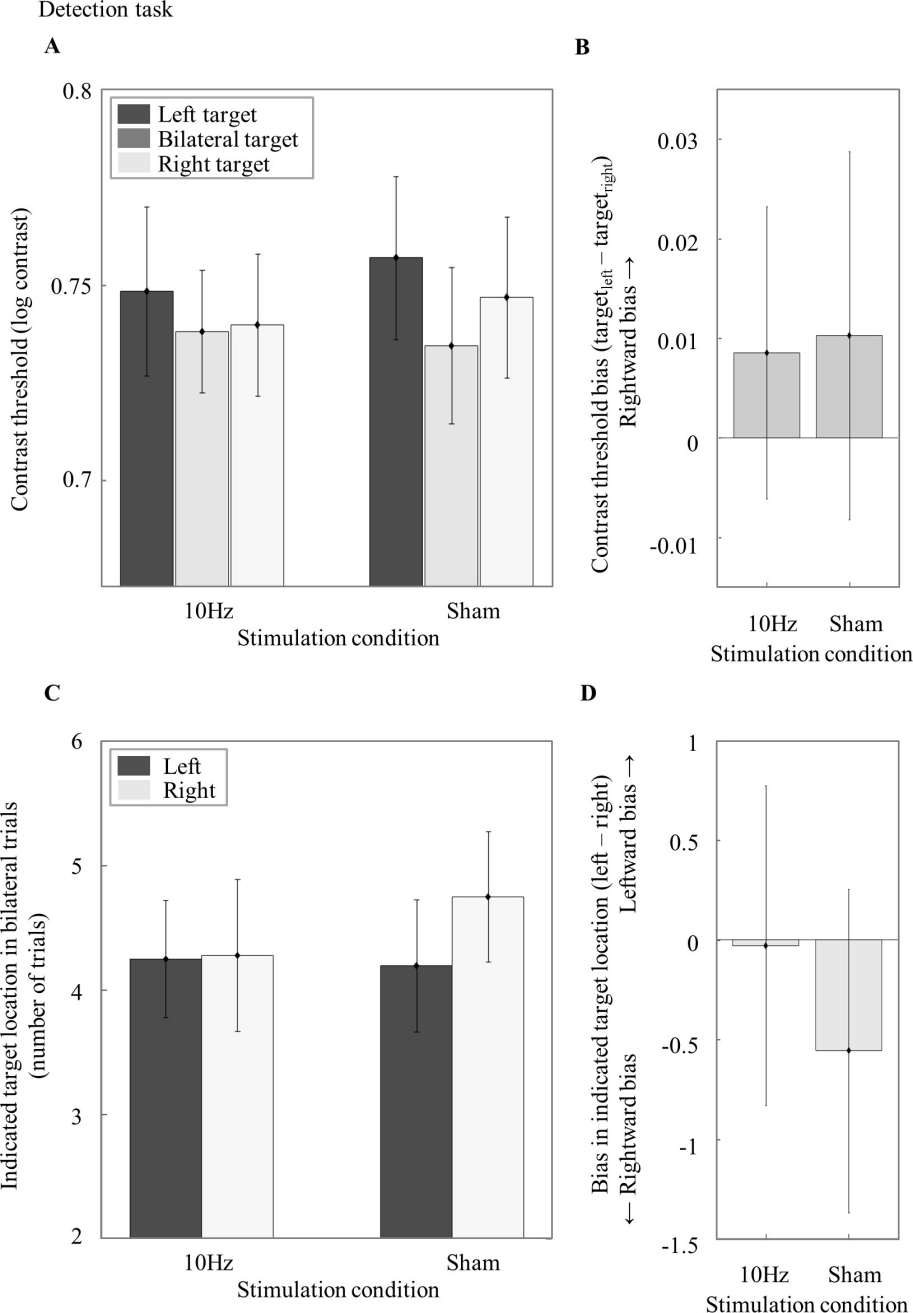
Results from the detection task. (A) Contrast thresholds in the detection task during 10Hz and sham tACS for left (dark grey), bilateral (grey) and right (light grey) targets. (B) *Contrast threshold bias (target*_*left*_*—target*_*right*_*) for the 10Hz tACS and sham Stimulation Condition*. (C) *Indicated Target Location (number of trials) in Bilateral Trials for the 10Hz and sham Stimulation Condition per left and right Indicated Target Location*. In some bilateral target trials, participants mistakenly indicated that there was only one single target stimulus in the left (left) or right hemifield (right). These errors can reveal biases in attentional selection in the context of multiple, simultaneously presented stimuli. (D) *Bias in Indicated Target Location (left—right) for the 10Hz and sham condition*. A positive and negative value stand for a right- and leftward bias respectively. The error bars visualize the standard error of the mean (SEM) across participants.

To investigate whether 10Hz tACS has an effect on contrast threshold and error bias in the detection task, we performed a repeated measures ANOVA with *Stimulation Condition* (10Hz, sham) and *Target Location* (left, right, bilateral) as factors and contrast thresholds as dependent variable. This analysis did not reveal a main effect of Stimulation Condition (F_1,35_ = .06, p = .813, ηp^2^ < .01), Target Location (F_2,70_ = .94, p = .397, ηp^2^ = .03) or an interaction effect (F_2,70_ = .284, p = .754, ηp^2^ = .01) (Fig 6A). We subsequently analyzed whether 10Hz tACS induced a contrast threshold bias in the detection task by comparing the contrast threshold bias score (target_left_—target_right_) of the two Stimulation Conditions in a paired-samples t-test. There was no significant difference between the 10Hz tACS (M = .01, SEM = .01) and the sham Stimulation Condition (M = .01, SEM = .02) (t_35_ = -.08, p = .94) (Fig 6B).

To test whether tACS at 10Hz also affected attentional selection we analyzed the number of incorrect responses in the bilateral trials, i.e. whether participants only perceived the left or the right stimulus when actually a bilateral stimulus was shown. Stimulation Condition and *Indicated Target Location in Bilateral Trials* (left, right) was added as factors in a repeated measures ANOVA and number of correct trials as dependent variable. There was no main effect of Stimulation Condition (F_1,35_ = .53, p = .471, ηp^2^ = .02) or Indicated Target Location in Bilateral Trials (F_1,35_ = .16, p = .695, ηp^2^ < .01) and no interaction effect (F_1,35_ = .64, p = .429, ηp^2^ = .02) (Fig 6C). We also analyzed with a paired-samples t-test whether there is any difference in error bias (response_left_—response_right_) between the two Stimulation Conditions. Also here, the 10Hz tACS condition (M = -.03, SEM = .80) did not differ from sham (M = -.56, SEM = .81) (t_35_ = .80, p = .429) (Fig 6D). This suggests that performance in the detection task was not affected by 10Hz tACS.

For a better comparison between tasks, we also post-hoc explored the reaction time bias (RTleft targets—RT_right targets_) in the detection task. We used a paired samples t-test to analyze the differences in reaction time bias between Stimulation Conditions. There was no significant difference between the 10Hz (M = 20.92, SEM = 13.42) and sham (M = -.63, SEM = 10.03) Stimulation Condition (t_35_ = 1.31, p = .199). To investigate whether 10Hz tACS induced a reaction time bias in the detection task, we also ran a repeated measures ANOVA with Target Location (left, right) and Stimulation Condition (10Hz, sham) as factors and median RTs as dependent variable. There was a main effect of Target Location (F_2,70_ = 5.27, p = .007), which suggests that participants had a baseline reaction time bias independent of the Stimulation Condition. However, we did not find an effect of Stimulation Condition (F_1,35_ = .15, p = .702) or interaction effect (F_2,70_ = 1.30, p = .278). This seems to indicate that the tACS intervention did not induce a reaction time bias in the detection task. While these analyses could be interpreted as further support for our results not being a simple bias in perception, it should again be noted that during the detection task, participants were instructed to focus on accuracy rather than response time.

### Blinding success

Throughout the experiment, participants were blinded to the experimental hypotheses and the stimulation protocol. At the end of each session, we administered a questionnaire, in which participants had to judge whether real or sham stimulation was applied. To assure that participants were not able to differentiate between the two *Stimulation Conditions* (10 Hz tACS, sham), we ran a generalized estimating equation analysis [50] with actual Stimulation Condition as factor and *Rated Stimulation Condition* as dependent variable (Table 1). Rated Stimulation Condition was assessed on an ordinal scale with seven levels. The value one corresponded to ‘I definitely experienced placebo/sham stimulation’ and the value seven to ‘I definitely experienced real stimulation’. According to the Wald chi square test, the actual Stimulation Condition did not affect the Rated Stimulation Condition (*X*^2^(1, N = 64) = .205, p = .651), indicating that blinding was indeed maintained.

**Table 1:** Outcomes of a post-stimulation questionnaire assessing the Rated Stimulation Condition based on the participant’s subjective experience.

		I definitely experienced sham stimulation	I most probably experienced sham stimulation	I might have experienced sham stimulation	I do not know	I might have experienced real stimulation	I most probably experienced real stimulation	I definitely experienced real stimulation
What kind of stimulation do you think you experienced today?	10Hz tACS	3.3%	23.3%	16.7%	16.7%	10%	23.3%	6.7%
	Sham	2.9%	23.5%	14.7%	32.4%	5.9%	14.7%	5.9%

Each cell indicates the percentage of responses per answer option and Stimulation Condition.

## Discussion

Previous EEG studies have shown an association between bias in attention and lateralization of parieto-occipital alpha power, showing greater power in the ipsilateral relative to the contralateral side of attention [24–27]. Here, we tested whether this association is robust enough to permit manipulations of the spatial distribution of attention by experimentally inducing hemispheric increases in oscillatory alpha power using tACS. We therefore stimulated the left parietal cortex with high-density tACS either at 10Hz or sham while assessing the bias in visuospatial attention with an endogenous and exogenous attention task, as well as a visuospatial detection task. The present report is (among) the first to show that visuospatial attention can be influenced by tACS at alpha frequency. In the endogenous attention task, a robust leftward bias was induced during 10Hz tACS as compared to sham. Interestingly, no significant stimulation effects were found in the detection task and exogenous attention task, indicating a task-specificity of the left parietal tACS intervention.

### Task specific tACS effects

tACS induced a spatial attention bias in the endogenous but not in the exogenous attention task and there are various explanations for this task-specific stimulation effect. We applied tACS to the posterior parietal cortex (PPC), a key area for the direction of attention towards a location of interest [39,40]. The PPC includes the intraparietal sulcus (IPS) an important node of the dorsal attention network (DAN). This site is generally associated with the endogenous control of attention [39,51,52], which might account for the robust tACS effect in the endogenous attention task. Moreover, various EEG studies have shown a link between a bias in endogenous attention and parieto-occipital alpha power lateralization [24–27]. After presentation of an endogenous cue, a contralateral decrease [27] and an ipsilateral alpha power increase [53] can be observed. This commonly reported lateralization of alpha power is observed after the presentation of an endogenous cue but before a target stimulus is shown [24–27]. The anticipatory change in hemispheric alpha power prior to target onset speaks in favour of an endogenous rather than an exogenous attention process. This might explain why we found a stimulation effect in the endogenous but not in the exogenous attention task. An alternative explanation for the absence of tACS effects in the exogenous attention tasks lies in the nature of the exogenous cues. It is possible that the exogenous cueing effects simply outweighed the tACS effect. It has previously been shown that voluntary orienting can be interrupted by salient lateralized cues [54–56]. Accordingly, the tACS induced endogenous attention bias/reaction time bias (RT_left target_—RT_right targets_) in our experiment may have been overruled by the exogenous cues.

Analyzing the contrast threshold bias, attentional selection bias and reaction time bias in the detection task, there was also no evidence for a leftward bias during 10Hz tACS as compared to sham. Our findings confirm the results of a recent transcranial current stimulation (tDCS) study reporting no effect of posterior parietal tDCS on contrast thresholds [3]. In contrast to the endogenous and exogenous attention tasks, which involved an attentional manipulation with cues, the detection task simply measured low-level perceptual sensitivity without such manipulation of the attentional focus. Moreover, the detection task required the participants to simply detect the target and indicates its approximate location whereas the endogenous and exogenous attention tasks required the participant to discriminate the target´s orientation. It could be argued that left parietal tACS did not affect lower-level visual processing such as target detection performance but rather higher-level attentional processes. This is in line with an fMRI study showing that the PPC is active during the voluntary direction of attention while target detection is rather regulated by the temporoparietal junction [39]. Alternatively, it could be argued that our detection task was simply not sensitive enough to measure the visuospatial attention bias induced by tACS at alpha frequency. In contradiction to our finding, it has previously been shown that within and between subject target detection performance is associated with pre-stimulus alpha power [57,58]. However, in those experiments, the change in alpha power was found at parieto-occipital electrode sites. The occipital cortex mainly processes sensory input, i.e. features of visual stimuli such as orientation, spatial frequency, color and movement [59–62]. Only a small part of the occipital cortex shows attentional modulation [63]. The fact that the association was also found at occipital sites might account for the link between alpha power and low-level target detection performance. In the current experiment we limited stimulation to the left PPC leaving out the occipital cortex. This might explain the absence of stimulation effects in the detection task in our experiment.

Hopfinger and colleagues [64] employed a similar study design stimulating the right parietal cortex with tACS at 10Hz, 40Hz or sham while participants performed an endogenous and an exogenous attention task. They report that stimulation at gamma frequency (40Hz) decreased RTs specifically in invalid cue trials of the exogenous attention task. In contrast, we found that 10Hz tACS induces a general spatial attention bias in the endogenous attention task and we can only speculate why there was no differential effect on the cue types. One explanation might lie in the state-dependency of tACS effects. tACS at alpha frequency has shown to successfully increase alpha power when participants keep their eyes open during stimulation [29,65]. In contrast, this effect is weakened or even absent when participants keep their eyes closed [29,65]. As eyes open resting states are associated with an alpha power suppression and activation of the occipital cortex [66,67], these electrophysiological measures might indicate a high susceptibility for alpha tACS effects. Various experiments show that endogenous cues lead to an alpha power suppression contralateral to the cued hemifield [26,27,68]. Moreover, valid, neutral as well as invalid endogenous cues lead to an activation of the PPC [69–71]. It is plausible that the alpha power suppression and the overall activation of our stimulation site in the cue target interval increased its susceptibility for neuromodulation. This in turn might have led to the general, cue-independent stimulation effect in the endogenous attention task. Contrary to this interpretation, other sources suggest that tACS requires an involvement rather than a suppression of the to-be stimulated oscillation in order to be effective [72,73]. Kasten and colleagues [72] argue that tACS seems to modulate elevated pre-stimulus alpha power rather than suppressed post-stimulus alpha power. However, this assumption was never explicitly tested because the authors limited their analysis to a difference score between pre- and post-stimulus alpha power in order to account for tACS artefacts. Their results show a baseline difference between pre- and post-stimulus alpha power, with higher power before as compared to after presentation of the visual stimulus. Application of tACS at alpha frequency in turn led to an amplification of this event-related power difference. The authors inferred from this finding that tACS presumably enhanced the already elevated pre-stimulus alpha power instead of suppressing post-stimulus alpha power. This interpretation is in line with a similar experiment showing the same offline stimulation effect [73] but further direct evidence is needed to confirm the assumption about the direction of the state-dependent tACS effects.

### Stimulation site and ring electrodes

Through left parietal tACS at 10Hz, we successfully shifted visuospatial attention to the left hemifield. In a similar attempt, Veniero and colleagues [38] conducted two consecutive studies in which they targeted the right parietal cortex with tACS at alpha frequency in order to induce a rightward bias. In experiment 1, they found the expected rightward bias for 10Hz tACS as compared to sham in a line bisection (landmark) task, but this finding could not be replicated in a second experiment. Likewise, Hopfinger and colleagues [64] administered right parietal tACS at alpha frequency and report no effect of 10Hz stimulation on spatial attention bias.

These results are surprising considering the rather established association of parietal alpha power lateralization with visuospatial attention. One aspect that distinguishes our experiment from the above-mentioned studies is that we used ring instead of disc electrodes. Compared to standard, rectangular, electrode configurations, ring electrodes enable a higher spatial focality [42], making it possible to limit stimulation to the left parietal cortex. Another difference compared the above-mentioned studies lies in the stimulation site. We stimulated the left instead of the right parietal cortex. A recent fMRI experiment including an endogenous as well as an exogenous attention task showed that task-related activity is greater in the left as compared to the right frontoparietal attention network [74]. Here, the left hemisphere seemed to be especially involved in reorienting, showing greater activation for invalid as compared to valid cue trials. Moreover, the change in functional connectivity during an endogenous attention task as compared to rest was shown to be more pronounced in the left as compared to the right hemisphere [75]. At rest, functional connectivity in the frontoparietal network was tonically higher in the right as compared to the left hemisphere. However, the left hemisphere was more specifically recruited during high attentional demands thereby balancing out the right hemispheric asymmetry. This might explain why left parietal tACS in our experiment induced a leftward bias in visuospatial attention whereas right parietal tACS has previously led to inconsistent results. Unfortunately, we are not able to draw any strong conclusions about the hemisphere specificity of our stimulation effect as we did not include the right parietal cortex as a control stimulation site. Future research should directly compare left and right hemispheric stimulation to investigate whether the functional control over endogenous spatial attention shifts is limited to the left parietal cortex.

### Clinical relevance, limitations and suggestions for future research

It must be noted that we cannot make strong claims about the frequency specificity of our stimulation effect as we did not include a control frequency or EEG measurements. Our findings are in line with the role of posterior parietal alpha oscillations in visuospatial attention [24–27] and are consistent with Wöstmann and colleagues [37] showing that unilateral tACS at alpha frequency leads to an ipsilateral shift of attention. Nevertheless, the question remains whether our behavioural tACS effect was caused by a direct modulation of alpha oscillations in the stimulated hemisphere or via indirect mechanisms such as phosphenes, broadband effects or cross-frequency coupling.

Phosphenes refer to the perception of light flashes without any changes of the external visual input entering the eye. They can be induced directly via alternating current stimulation of the retina [76] or indirectly via volume conduction from the visual cortex to the retina [77,78]. Kanai and colleagues [34] applied a distant-bipolar electrode montage over the visual cortex and report that participants perceived phosphenes during tACS at 10Hz. The authors argue that the electrical stimulation directly modulated the neural activity underneath the electrodes, which in turn elicited the phosphenes. However, this hypothesis has been challenged [77–79] and strong evidence points towards a retinal origin of the visual percept [77]. Opponents argue that the distant-bipolar tACS montage resulted in current spread from the occipital electrode to the retina, which led to voltage changes near the eye [77–79]. Finite element models are in line with this critique showing that distant-bipolar electrode montages produce diffuse, un-focal electrical fields. In contrast, concentric ring electrode montages, as used in this present experiment, result in spatially focal electrical fields. The results of our current simulation (Fig 1A) indeed shows that the electrical field produced by our ring electrode montage centred on P3, was limited to the left parietal cortex. This spatially confined electrical field rules out any confounds that would be caused by an electrical stimulation of the retina.

The term broadband effect describes an oscillatory response which covers a wide range of frequencies. Repetitive transcranial magnetic stimulation (rTMS), a non-invasive brain stimulation technique which uses rhythmic electromagnetic pulses to modulate brain oscillations [80,81], has shown to induce such a broadband effect [81]. Tuning rTMS to the alpha frequency has previously led to an increase in delta, theta, alpha beta and gamma power [82–84]. This is problematic from a theoretical point of view because it means that no strong conclusions about frequency-specificity of the effects can be drawn. In contrast, the sinusoidal current of tACS is bound to one frequency and therefore less likely to induce a broadband response [85]. Several experiments have shown that tACS at 10Hz leads to a specific power increase in the alpha frequency spectrum [29,30,86,87]. Alternating current stimulation induces a sinusoidal modulation of the membrane potential, which in turn influences spike timing in neural networks [88–90]. In vivo recordings in ferrets and non-human primates show that even small alternating currents on the surface of the skull can lead to a synchronization of neural firing to the phase of the applied sinusoidal current [89,91]. Although we cannot rule out this alternative explanation, evidence speaks against such a broadband effect in our experiment.

Another alternative explanation for our behavioural stimulation effect is cross-frequency coupling. It is theoretically possible that tACS at alpha frequency modulated gamma power via the inverse gamma-alpha power relationship [92–94]. Consequently, left parietal stimulation at alpha frequency would have led to a decrease in gamma power in the stimulated hemisphere. Gamma oscillations are associated with the conscious perception of stimuli with a power increase when attention is directed to a visual stimulus [95–97] and they are thus also functionally inversely related to alpha oscillations. As such, lower gamma power in the left as compared to the right hemisphere would have led to the same behavioural effect, which is a spatial attentional leftward bias. It has previously been shown that tACS at alpha frequency can lead to a decrease in gamma power [98]. It is therefore theoretically possible, that our behavioural stimulation effect was caused by a hemispheric change in gamma instead of alpha power. To strengthen our conclusion of an alpha-frequency specific stimulation effect, future research should include a control stimulation frequency condition and measure pre- and post-stimulation EEG to assure that alpha power was indeed boosted.

The possibility of modulating visuospatial attention through tACS at alpha frequency is not only relevant in the framework of fundamental research but might also have implications for the treatment of hemineglect patients. Common rehabilitation treatments for neglect patients focus on the contralesional enhancement of attention [99] through e.g. prism adaptation [100] or vestibular stimulation [101,102]. Recently, transcranial magnetic stimulation approaches have been introduced for non-invasively disrupting the unaffected hemisphere and thereby alleviating neglect symptoms [103,104]. In the present report, we showed that it is possible to induce a visuospatial attention bias in healthy participants through unilateral parietal tACS at alpha frequency. It remains to be seen whether tACS at alpha frequency can also be used to treat patients with attentional deficits. Hemineglect patients commonly suffer from a pathological rightward bias [6,7]. This rightward bias could be counteracted with left parietal tACS at alpha frequency, as demonstrated here with healthy participants. tACS has been proposed to induce neuroplastic changes under the stimulation site [85,86,105]. This might make it a potential easy-to-apply, portable and affordable treatment for hemineglect patients with long-term benefits [105].

## References

[1] Posner MI. Orienting of attention. Q J Exp Psychol. 1980;32: 3–25. 10.1080/00335558008248231 7367577

[2] Kim H, Levine SC. Sources of between-subjects variability in perceptual asymmetries: A meta-analytic review. Neuropsychologia. 1991;29: 877–888. 10.1016/0028-3932(91)90053-b 1834960

[3] Duecker F, Schuhmann T, Bien N, Jacobs C, Sack AT. Moving Beyond Attentional Biases: Shifting the Interhemispheric Balance between Left and Right Posterior Parietal Cortex Modulates Attentional Control Processes. J Cogn Neurosci. 2017;29: 1267–1278. 10.1162/jocn_a_01119 28294715

[4] Shepherd M, Müller HJ. Movement versus focusing of visual attention. Percept Psychophys. 1989; 10.3758/BF03204974 2762102

[5] Cheal M Lou Lyon DR. Central and Peripheral Precuing of Forced-choice Discrimination. Q J Exp Psychol Sect A. 1991; 10.1080/14640749108400960 1775667

[6] Buxbaum LJ, Ferraro MK, Veramonti T, Farne A, Whyte J, Ladavas E, et al Hemispatial neglect: Subtypes, neuroanatomy, and disability. Neurology. 2004;62: 749–756. 10.1212/01.wnl.0000113730.73031.f4 15007125

[7] Ringman JM, Saver JL, Woolson RF, Clarke WR, Adams HP. Frequency, risk factors, anatomy, and course of unilateral neglect in an acute stroke cohort. Neurology. 2004;63: 468–474. 10.1212/01.wnl.0000133011.10689.ce 15304577

[8] Vallar G. Spatial hemineglect in humans. Prog Neurobiol. 1998; 10.1016/S0301-0082(00)00028-9 21227084

[9] Smania N. The spatial distribution of visual attention in hemineglect and extinction patients. Brain. 1998;121: 1759–1770. 10.1093/brain/121.9.1759 9762963

[10] Marzi CA, Natale E, Anderson B. Mapping spatial attention with reaction time in neglect patients The Cognitive and Neural Bases of Spatial Neglect. Oxford University Press; 2002 pp. 274–288. 10.1093/acprof:oso/9780198508335.003.0020

[11] Bartolomeo P, Sieroff E, Decaix C, Chokron S. Modulating the attentional bias in unilateral neglect: the effects of the strategic set. Exp Brain Res. 2001;137: 432–444. 10.1007/s002210000642 11355388

[12] Morrow LA, Ratcliff G. The disengagement of covert attention and the neglect syndrome. Psychobiology. 1988;16: 261–269. 10.3758/BF03327316

[13] Posner MI, Cohen Y. Components of visual orienting. J Cogn Neurosci. 1984;32: 531–556. 10.1162/jocn.1991.3.4.335 23967813

[14] Kerkhoff G. Spatial hemineglect in humans. Progress in Neurobiology. 2001 10.1016/S0301-0082(00)00028-9 11040416

[15] Ros T, Michela A, Bellman A, Vuadens P, Saj A, Vuilleumier P. Increased Alpha-Rhythm Dynamic Range Promotes Recovery from Visuospatial Neglect: A Neurofeedback Study. Neural Plast. 2017;2017: 1–9. 10.1155/2017/7407241 28529806 PMC5424484

[16] Finnigan S, van Putten MJAM. EEG in ischaemic stroke: Quantitative EEG can uniquely inform (sub-)acute prognoses and clinical management. Clin Neurophysiol. 2013;124: 10–19. 10.1016/j.clinph.2012.07.003 22858178

[17] Sainio K, Stenberg D, Keskimäki I, Muuronen A, Kaste M. Visual and spectral EEG analysis in the evaluation of the outcome in patients with ischemic brain infarction. Electroencephalogr Clin Neurophysiol. 1983;56: 117–124. 10.1016/0013-4694(83)90066-4 6191943

[18] Giaquinto S, Cobianchi A, Macera F, Nolfe G. EEG recordings in the course of recovery from stroke. Stroke. 1994;25: 2204–2209. 10.1161/01.str.25.11.2204 7974546

[19] de Weerd AW, Veldhuizen RJ, Veering MM, Poortvliet DCJ, Jonkman EJ. Recovery from cerebral ischaemia. EEG, cerebral blood flow and clinical symptomatology in the first three years after a stroke. Electroencephalogr Clin Neurophysiol. 1988;70: 197–204. 10.1016/0013-4694(88)90080-6 2458226

[20] Szelies B, Mielke R, Kessler J, Heiss W-D. Prognostic relevance of quantitative topographical EEG in patients with poststroke aphasia. Brain Lang. 2002;82: 87–94. 10.1016/s0093-934x(02)00004-4 12174818

[21] Başar E, Başar-Eroglu C, Karakaş S, Schürmann M. Are cognitive processes manifested in event-related gamma, alpha, theta and delta oscillations in the EEG? Neurosci Lett. 1999; 10.1016/S0304-3940(98)00934-3 10025584

[22] Klimesch W. Alpha-band oscillations, attention, and controlled access to stored information. Trends Cogn Sci. 2012;16: 606–617. 10.1016/j.tics.2012.10.007 23141428 PMC3507158

[23] Klimesch W, Sauseng P, Hanslmayr S. EEG alpha oscillations: The inhibition-timing hypothesis. Brain Research Reviews. 2007 10.1016/j.brainresrev.2006.06.003 16887192

[24] Gould IC, Rushworth MF, Nobre AC. Indexing the graded allocation of visuospatial attention using anticipatory alpha oscillations. J Neurophysiol. 2011;105: 1318–1326. 10.1152/jn.00653.2010 21228304 PMC3074422

[25] Händel BF, Haarmeier T, Jensen O. Alpha Oscillations Correlate with the Successful Inhibition of Unattended Stimuli. J Cogn Neurosci. 2011;23: 2494–2502. 10.1162/jocn.2010.21557 20681750

[26] Sauseng P, Klimesch W, Stadler W, Schabus M, Doppelmayr M, Hanslmayr S, et al A shift of visual spatial attention is selectively associated with human EEG alpha activity. Eur J Neurosci. 2005;22: 2917–2926. 10.1111/j.1460-9568.2005.04482.x 16324126

[27] Thut G, Nietzel A, Brandt SA, Pascual-Leone A. -Band Electroencephalographic Activity over Occipital Cortex Indexes Visuospatial Attention Bias and Predicts Visual Target Detection. J Neurosci. 2006;26: 9494–9502. 10.1523/JNEUROSCI.0875-06.2006 16971533 PMC6674607

[28] Antal A, Paulus W. Transcranial Alternating Current Stimulation. Front Hum Neurosci. 2013;7: 317 10.3389/fnhum.2013.00317 23825454 PMC3695369

[29] Neuling T, Rach S, Herrmann CS. Orchestrating neuronal networks: sustained after-effects of transcranial alternating current stimulation depend upon brain states. Front Hum Neurosci. 2013;7: 161 10.3389/fnhum.2013.00161 23641206 PMC3639376

[30] Zaehle T, Rach S, Herrmann CS. Transcranial Alternating Current Stimulation Enhances Individual Alpha Activity in Human EEG. Aleman A, editor. PLoS One. 2010;5: e13766 10.1371/journal.pone.0013766 21072168 PMC2967471

[31] Kasten FH, Dowsett J, Herrmann CS. Sustained Aftereffect of α-tACS Lasts Up to 70 min after Stimulation. Front Hum Neurosci. 2016;10: 245 10.3389/fnhum.2016.00245 27252642 PMC4879138

[32] Witkowski M, Garcia-Cossio E, Chander BS, Braun C, Birbaumer N, Robinson SE, et al Mapping entrained brain oscillations during transcranial alternating current stimulation (tACS). Neuroimage. 2016;140: 89–98. 10.1016/j.neuroimage.2015.10.024 26481671

[33] Feurra M, Paulus W, Walsh V, Kanai R. Frequency Specific Modulation of Human Somatosensory Cortex. Front Psychol. 2011;2: 13 10.3389/fpsyg.2011.00013 21713181 PMC3111335

[34] Kanai R, Chaieb L, Antal A, Walsh V, Paulus W. Frequency-Dependent Electrical Stimulation of the Visual Cortex. Curr Biol. 2008;18: 1839–1843. 10.1016/j.cub.2008.10.027 19026538

[35] Schwiedrzik C. Retina or visual cortex? The site of phosphene induction by transcranial alternating current stimulation. Front Integr Neurosci. 2009;3 10.3389/neuro.07.006.2009 19506706 PMC2691656

[36] Wach C, Krause V, Moliadze V, Paulus W, Schnitzler A, Pollok B. Effects of 10Hz and 20Hz transcranial alternating current stimulation (tACS) on motor functions and motor cortical excitability. Behav Brain Res. 2013;241: 1–6. 10.1016/j.bbr.2012.11.038 23219965

[37] Wöstmann M, Vosskuhl J, Obleser J, Herrmann CS. Opposite effects of lateralised transcranial alpha versus gamma stimulation on auditory spatial attention. Brain Stimul. 2018;11: 752–758. 10.1016/j.brs.2018.04.006 29656907

[38] Veniero D, Benwell CSY, Ahrens MM, Thut G. Inconsistent Effects of Parietal α-tACS on Pseudoneglect across Two Experiments: A Failed Internal Replication. Front Psychol. 2017;8: 952 10.3389/fpsyg.2017.00952 28642729 PMC5463322

[39] Corbetta M, Kincade JM, Ollinger JM, McAvoy MP, Shulman GL. Voluntary orienting is dissociated from target detection in human posterior parietal cortex. Nat Neurosci. 2000;3: 292–297. 10.1038/73009 10700263

[40] Yantis S, Schwarzbach J, Serences JT, Carlson RL, Steinmetz MA, Pekar JJ, et al Transient neural activity in human parietal cortex during spatial attention shifts. Nat Neurosci. 2002; 10.1038/nn921 12219097

[41] Antal A, Alekseichuk I, Bikson M, Brockmöller J, Brunoni AR, Chen R, et al Low intensity transcranial electric stimulation: Safety, ethical, legal regulatory and application guidelines. Clin Neurophysiol. 2017;128: 1774–1809. 10.1016/j.clinph.2017.06.001 28709880 PMC5985830

[42] Datta A, Elwassif M, Battaglia F, Bikson M. Transcranial current stimulation focality using disc and ring electrode configurations: FEM analysis. J Neural Eng. 2008;5: 163–174. 10.1088/1741-2560/5/2/007 18441418

[43] Heise KF, Monteiro TS, Leunissen I, Mantini D, Swinnen SP. Distinct online and offline effects of alpha and beta transcranial alternating current stimulation (tACS) on continuous bimanual performance and task-set switching. Sci Rep. 2019; 10.1038/s41598-019-39900-0 30816305 PMC6395614

[44] Saturnino GB, Thielscher A, Madsen KH, Knösche TR, Weise K. A principled approach to conductivity uncertainty analysis in electric field calculations. Neuroimage. 2019;188: 821–834. 10.1016/j.neuroimage.2018.12.053 30594684

[45] Saturnino GB, Puonti O, Nielsen JD, Antonenko D, Madsen KHH, Thielscher A. SimNIBS 2.1: A Comprehensive Pipeline for Individualized Electric Field Modelling for Transcranial Brain Stimulation. bioRxiv. 2018; 10.1101/500314 31725247

[46] Boayue NM, Csifcsák G, Puonti O, Thielscher A, Mittner M. Head models of healthy and depressed adults for simulating the electric fields of non-invasive electric brain stimulation. F1000Research. 2018;7: 704 10.12688/f1000research.15125.2 30505431 PMC6241565

[47] Saturnino GB, Antunes A, Thielscher A. On the importance of electrode parameters for shaping electric field patterns generated by tDCS. Neuroimage. 2015; 10.1016/j.neuroimage.2015.06.067 26142274

[48] Watson AB, Pelli DG. Quest: A Bayesian adaptive psychometric method. Percept Psychophys. 1983;33: 113–120. 10.3758/bf03202828 6844102

[49] Brainard DH. The Psychophysics Toolbox. Spat Vis. 1997; 10.1163/156856897X00357 9176952

[50] Liu X, Zhang J. Analysis of ordinal repeated measures data using generalized estimating equation. Sichuan Da Xue Xue Bao Yi Xue Ban. 2006;37: 798–800. 17037756

[51] Kincade JM. An Event-Related Functional Magnetic Resonance Imaging Study of Voluntary and Stimulus-Driven Orienting of Attention. J Neurosci. 2005; 10.1523/jneurosci.0236-05.2005 15872107 PMC6725019

[52] Ozaki TJ. Frontal-to-parietal top-down causal streams along the dorsal attention network exclusively mediate voluntary orienting of attention. PLoS One. 2011; 10.1371/journal.pone.0020079 21611155 PMC3096666

[53] Worden MS, Foxe JJ, Wang N, Simpson G V. Anticipatory Biasing of Visuospatial Attention Indexed by Retinotopically Specific α-Bank Electroencephalography Increases over Occipital Cortex. J Neurosci. 2018; 10.1523/jneurosci.20-06-j0002.2000 10704517 PMC6772495

[54] Müller HJ, Rabbitt PMA. Reflexive and voluntary orienting of visual attention: Time course of activation and resistance to interruption. J Exp Psychol Hum Percept Perform. 1989;15: 315–330. 10.1037//0096-1523.15.2.315 2525601

[55] van der Lubbe RHJ, Postma A. Interruption from irrelevant auditory and visual onsets even when attention is in a focused state. Exp Brain Res. 2005;164: 464–471. 10.1007/s00221-005-2267-0 15785951

[56] Yantis S, Jonides J. Abrupt Visual Onsets and Selective Attention: Voluntary Versus Automatic Allocation. J Exp Psychol Hum Percept Perform. 1990;16: 121–134. 10.1037//0096-1523.16.1.121 2137514

[57] Ergenoglu T, Demiralp T, Bayraktaroglu Z, Ergen M, Beydagi H, Uresin Y. Alpha rhythm of the EEG modulates visual detection performance in humans. Brain Res Cogn Brain Res. 2004;20: 376–383. 10.1016/j.cogbrainres.2004.03.009 15268915

[58] Hanslmayr S, Aslan A, Staudigl T, Klimesch W, Herrmann CS, Bäuml K-H. Prestimulus oscillations predict visual perception performance between and within subjects. Neuroimage. 2007;37: 1465–1473. 10.1016/j.neuroimage.2007.07.011 17706433

[59] Shipp S, Zeki S. Segregation and convergence of specialised pathways in macaque monkey visual cortex. J Anat. 1995;187: 547–562. 8586555 PMC1167459

[60] Sincich LC. Input to V2 Thin Stripes Arises from V1 Cytochrome Oxidase Patches. J Neurosci. 2005;25: 10087–10093. 10.1523/JNEUROSCI.3313-05.2005 16267215 PMC6725776

[61] Bartels A, Zeki S. The architecture of the colour centre in the human visual brain: new results and a review *. Eur J Neurosci. 2000;12: 172–193. 10.1046/j.1460-9568.2000.00905.x 10651872

[62] Brewer AA, Liu J, Wade AR, Wandell BA. Visual field maps and stimulus selectivity in human ventral occipital cortex. Nat Neurosci. 2005;8: 1102–1109. 10.1038/nn1507 16025108

[63] Ghose GM, Maunsell JHR. Attentional modulation in visual cortex depends on task timing. Nature. 2002;419: 616–620. 10.1038/nature01057 12374979

[64] Hopfinger JB, Parsons J, Fröhlich F. Differential effects of 10-Hz and 40-Hz transcranial alternating current stimulation (tACS) on endogenous versus exogenous attention. Cogn Neurosci. 2017;8: 102–111. 10.1080/17588928.2016.1194261 27297977

[65] Ruhnau P, Neuling T, Fuscá M, Herrmann CS, Demarchi G, Weisz N. Eyes wide shut: Transcranial alternating current stimulation drives alpha rhythm in a state dependent manner. Sci Rep. 2016;6: 27138 10.1038/srep27138 27252047 PMC4890046

[66] Marx E, Deutschländer A, Stephan T, Dieterich M, Wiesmann M, Brandt T. Eyes open and eyes closed as rest conditions: Impact on brain activation patterns. Neuroimage. 2004; 10.1016/j.neuroimage.2003.12.026 15050602

[67] Adrian ED, Matthews BHC. The berger rhythm: Potential changes from the occipital lobes in man. Brain. 1934; 10.1093/brain/57.4.355 20058345

[68] Capilla A, Schoffelen J-M, Paterson G, Thut G, Gross J. Dissociated α-Band Modulations in the Dorsal and Ventral Visual Pathways in Visuospatial Attention and Perception. Cereb Cortex. 2014;24: 550–561. 10.1093/cercor/bhs343 23118197 PMC3888375

[69] Kim Y-H, Gitelman DR, Parrish TB, Nobre AC, LaBar KS, Mesulam M-M. Posterior Cingulate Activation Varies According to the Effectiveness of Attentional Engagement. Neuroimage. 2018; 10.1016/s1053-8119(18)30900-5

[70] Hopf J-M, Mangun G. Shifting visual attention in space: an electrophysiological analysis using high spatial resolution mapping. Clin Neurophysiol. 2000;111: 1241–1257. 10.1016/s1388-2457(00)00313-8 10880800

[71] Kato C, Matsuo K, Matsuzawa M, Moriya T, Glover GH, Nakai T. Activation during endogenous orienting of visual attention using symbolic pointers in the human parietal and frontal cortices: a functional magnetic resonance imaging study. Neurosci Lett. 2001;314: 5–8. 10.1016/s0304-3940(01)02207-8 11698133

[72] Kasten FH, Maess B, Herrmann CS. Facilitated Event-Related Power Modulations during Transcranial Alternating Current Stimulation (tACS) Revealed by Concurrent tACS-MEG. eneuro. 2018;5 10.1523/ENEURO.0069-18.2018 30073188 PMC6070188

[73] Kasten FH, Herrmann CS. Transcranial Alternating Current Stimulation (tACS) Enhances Mental Rotation Performance during and after Stimulation. Front Hum Neurosci. 2017;11: 2 10.3389/fnhum.2017.00002 28197084 PMC5281636

[74] Meyer KN, Du F, Parks E, Hopfinger JB. Exogenous vs. endogenous attention: Shifting the balance of fronto-parietal activity. Neuropsychologia. 2018;111: 307–316. 10.1016/j.neuropsychologia.2018.02.006 29425803

[75] Meehan TP, Bressler SL, Tang W, Astafiev S V., Sylvester CM, Shulman GL, et al Top-down cortical interactions in visuospatial attention. Brain Struct Funct. 2017;222: 3127–3145. 10.1007/s00429-017-1390-6 28321551 PMC5607080

[76] Brindley GS. The site of electrical excitation of the human eye. J Physiol. 1955;127: 189–200. 10.1113/jphysiol.1955.sp005248 14354638 PMC1365848

[77] Kar K, Krekelberg B. Transcranial electrical stimulation over visual cortex evokes phosphenes with a retinal origin. J Neurophysiol. 2012; 10.1152/jn.00505.2012 22855777 PMC3545027

[78] Schutter DJLG, Hortensius R. Retinal origin of phosphenes to transcranial alternating current stimulation. Clin Neurophysiol. 2010; 10.1016/j.clinph.2009.10.038 20188625

[79] Laakso I, Hirata A. Computational analysis shows why transcranial alternating current stimulation induces retinal phosphenes. J Neural Eng. 2013; 10.1088/1741-2560/10/4/046009 23813466

[80] Herrmann CS, Strüber D, Helfrich RF, Engel AK. EEG oscillations: From correlation to causality. Int J Psychophysiol. 2016;103: 12–21. 10.1016/j.ijpsycho.2015.02.003 25659527

[81] Veniero D, Vossen A, Gross J, Thut G. Lasting EEG/MEG Aftereffects of Rhythmic Transcranial Brain Stimulation: Level of Control Over Oscillatory Network Activity. Front Cell Neurosci. 2015;9 10.3389/fncel.2015.00477 26696834 PMC4678227

[82] Okamura H, Jing H, Takigawa M. EEG Modification Induced by Repetitive Transcranial Magnetic Stimulation. J Clin Neurophysiol. 2001;18: 318–325. 10.1097/00004691-200107000-00003 11673697

[83] Woźniak-Kwaśniewska A, Szekely D, Aussedat P, Bougerol T, David O. Changes of oscillatory brain activity induced by repetitive transcranial magnetic stimulation of the left dorsolateral prefrontal cortex in healthy subjects. Neuroimage. 2014;88: 91–99. 10.1016/j.neuroimage.2013.11.029 24269574

[84] Griškova I, Rukšėnas O, Dapšys K, Herpertz S, Höppner J. The effects of 10Hz repetitive transcranial magnetic stimulation on resting EEG power spectrum in healthy subjects. Neurosci Lett. 2007;419: 162–167. 10.1016/j.neulet.2007.04.030 17478041

[85] Herrmann CS, Rach S, Neuling T, Strüber D. Transcranial alternating current stimulation: a review of the underlying mechanisms and modulation of cognitive processes. Front Hum Neurosci. 2013;7: 279 10.3389/fnhum.2013.00279 23785325 PMC3682121

[86] Vossen A, Gross J, Thut G. Alpha Power Increase After Transcranial Alternating Current Stimulation at Alpha Frequency (α-tACS) Reflects Plastic Changes Rather Than Entrainment. Brain Stimul. 2015;8: 499–508. 10.1016/j.brs.2014.12.004 25648377 PMC4464304

[87] Helfrich RF, Schneider TR, Rach S, Trautmann-Lengsfeld SA, Engel AK, Herrmann CS. Entrainment of brain oscillations by transcranial alternating current stimulation. Curr Biol. 2014; 10.1016/j.cub.2013.12.041 24461998

[88] Chan CY, Nicholson C. Modulation by applied electric fields of Purkinje and stellate cell activity in the isolated turtle cerebellum. J Physiol. 1986;371: 89–114. 10.1113/jphysiol.1986.sp015963 3701658 PMC1192712

[89] Ozen S, Sirota A, Belluscio MA, Anastassiou CA, Stark E, Koch C, et al Transcranial Electric Stimulation Entrains Cortical Neuronal Populations in Rats. J Neurosci. 2010;30: 11476–11485. 10.1523/JNEUROSCI.5252-09.2010 20739569 PMC2937280

[90] Reato D, Rahman A, Bikson M, Parra LC. Low-Intensity Electrical Stimulation Affects Network Dynamics by Modulating Population Rate and Spike Timing. J Neurosci. 2010;30: 15067–15079. 10.1523/JNEUROSCI.2059-10.2010 21068312 PMC3500391

[91] Johnson M, Alekseichuk I, Krieg J, Doyle A, Yu Y, Vitek J, et al Dose-dependent effects of transcranial alternating current stimulation on spike timing in awake nonhuman primates. bioRxiv. 2019; 696344 10.1101/696344 PMC7467690 32917605

[92] Lorenz I, Müller N, Schlee W, Hartmann T, Weisz N. Loss of alpha power is related to increased gamma synchronization—A marker of reduced inhibition in tinnitus? Neurosci Lett. 2009;453: 225–228. 10.1016/j.neulet.2009.02.028 19429040

[93] Lundqvist M, Herman P, Lansner A. Theta and Gamma Power Increases and Alpha/Beta Power Decreases with Memory Load in an Attractor Network Model. J Cogn Neurosci. 2011;23: 3008–3020. 10.1162/jocn_a_00029 21452933

[94] Spaak E, Bonnefond M, Maier A, Leopold DA, Jensen O. Layer-Specific Entrainment of Gamma-Band Neural Activity by the Alpha Rhythm in Monkey Visual Cortex. Curr Biol. 2012;22: 2313–2318. 10.1016/j.cub.2012.10.020 23159599 PMC3528834

[95] Mueller M, Bosch J, Elbert T, Kreiter A, Sosa M, Sosa P, et al Visually induced gamma-band responses in human electroencephalographic activity ? a link to animal studies. Exp Brain Res. 1996; 112 10.1007/BF00227182 8951411

[96] Tallon-Baudry C, Bertrand O, Delpuech C, Pernier J. Oscillatory γ-Band (30–70 Hz) Activity Induced by a Visual Search Task in Humans. J Neurosci. 1997;17: 722–734. 10.1523/JNEUROSCI.17-02-00722.1997 8987794 PMC6573221

[97] Gruber T, Mueller MM, Keil A, Elbert T. Selective visual-spatial attention alters induced gamma band responses in the human EEG. Clin Neurophysiol. 1999;110: 2074–2085. 10.1016/s1388-2457(99)00176-5 10616112

[98] Wach C, Krause V, Moliadze V, Paulus W, Schnitzler A, Pollok B. The effect of 10 Hz transcranial alternating current stimulation (tACS) on corticomuscular coherence. Front Hum Neurosci. 2013;7 10.3389/fnhum.2013.00511 24009573 PMC3756226

[99] Kerkhoff G, Schenk T. Rehabilitation of neglect: An update. Neuropsychologia. 2012;50: 1072–1079. 10.1016/j.neuropsychologia.2012.01.024 22306520

[100] Rossetti Y, Rode G, Pisella L, Farné A, Li L, Boisson D, et al Prism adaptation to a rightward optical deviation rehabilitates left hemispatial neglect. Nature. 1998;395: 166–169. 10.1038/25988 9744273

[101] Cappa S, Sterzi R, Vallar G, Bisiach E. Remission of hemineglect and anosognosia during vestibular stimulation. Neuropsychologia. 1987;25: 775–782. 10.1016/0028-3932(87)90115-1 3501552

[102] Rubens AB. Caloric stimulation and unilateral visual neglect. Neurology. 1985;35: 1019–1019. 10.1212/wnl.35.7.1019 4010940

[103] Fierro B, Brighina F, Bisiach E. Improving Neglect by TMS. Behav Neurol. 2006;17: 169–176. 10.1155/2006/465323 17148837 PMC5471542

[104] Oliveri M, Bisiach E, Brighina F, Piazza A, La Bua V, Buffa D, et al rTMS of the unaffected hemisphere transiently reduces contralesional visuospatial hemineglect. Neurology. 2001;57: 1338–1340. 10.1212/wnl.57.7.1338 11591865

[105] Antal A, Paulus W. Investigating Neuroplastic Changes in the Human Brain Induced by Transcranial Direct (tDCS) and Alternating Current (tACS) Stimulation Methods. Clin EEG Neurosci. 2012;43: 175–175. 10.1177/1550059412448030 22956645

